# Vessel Wall Magnetic Resonance Imaging in Cerebrovascular Diseases

**DOI:** 10.3390/diagnostics12020258

**Published:** 2022-01-20

**Authors:** Federico Mazzacane, Valentina Mazzoleni, Elisa Scola, Sara Mancini, Ivano Lombardo, Giorgio Busto, Elisa Rognone, Anna Pichiecchio, Alessandro Padovani, Andrea Morotti, Enrico Fainardi

**Affiliations:** 1 Department of Emergency Neurology and Stroke Unit, IRCCS Mondino Foundation, 27100 Pavia, Italy; federico.mazzacane01@universitadipavia.it; 2 Department of Brain and Behavioral Sciences, University of Pavia, 27100 Pavia, Italy; anna.pichiecchio@mondino.it; 3 Neurology Unit, Department of Clinical and Experimental Sciences, University of Brescia, 25121 Brescia, Italy; v.mazzoleni001@unibs.it (V.M.); alessandro.padovani@unibs.it (A.P.); 4 Neurology Unit, Department of Neurological Sciences and Vision, ASST-Spedali Civili, 25123 Brescia, Italy; andrea.morotti85@gmail.com; 5 Neuroradiology Unit, Department of Radiology, Careggi University Hospital, 50134 Florence, Italy; elisascola80@gmail.com (E.S.); saraman85@gmail.com (S.M.); ivano.lombardo@gmail.com (I.L.); giorgio.busto@libero.it (G.B.); 6 Department of Neuroradiology, IRCCS Mondino Foundation, 27100 Pavia, Italy; elisa.rognone@mondino.it; 7 Neuroradiology Unit, Department of Experimental and Clinical Biomedical Sciences “Mario Serio”, University of Florence, 50121 Florence, Italy

**Keywords:** cerebrovascular disease, vessel wall MRI, intracranial vasculopathy, stroke, atherosclerosis, vasculitis, dissection

## Abstract

Cerebrovascular diseases are a leading cause of disability and death worldwide. The definition of stroke etiology is mandatory to predict outcome and guide therapeutic decisions. The diagnosis of pathological processes involving intracranial arteries is especially challenging, and the visualization of intracranial arteries’ vessel walls is not possible with routine imaging techniques. Vessel wall magnetic resonance imaging (VW-MRI) uses high-resolution, multiparametric MRI sequences to directly visualize intracranial arteries walls and their pathological alterations, allowing a better characterization of their pathology. VW-MRI demonstrated a wide range of clinical applications in acute cerebrovascular disease. Above all, it can be of great utility in the differential diagnosis of atherosclerotic and non-atherosclerotic intracranial vasculopathies. Additionally, it can be useful in the risk stratification of intracranial atherosclerotic lesions and to assess the risk of rupture of intracranial aneurysms. Recent advances in MRI technology made it more available, but larger studies are still needed to maximize its use in daily clinical practice.

## 1. Introduction

Cerebrovascular diseases are a leading cause of disability and death worldwide. The definition of stroke etiology is mandatory to predict the prognosis and recurrence risk and guide therapeutic decisions [1,2]. The pathogenesis of cerebrovascular diseases often originates inside the vessel walls, therefore, standard luminal based imaging modalities lack the capability to visualize these pathological processes, unless they are associated with significant luminal changes [3]. This is particularly true for intracranial arteries, which have smaller diameter and thinner vessel walls, that are beyond the resolution of standard diagnostic techniques [4]. Vessel wall magnetic resonance imaging (VW-MRI) uses high-resolution, multiparametric MRI sequences to directly visualize intracranial arteries walls and their pathological alterations [5,6].

VW-MRI has demonstrated its utility in a wide range of clinical situations, including differential diagnosis of intracranial vasculopathies, identification and evaluation of intracranial atherosclerotic disease and also risk stratification in patients with intracranial aneurysms [7,8]. When applied to patients with cryptogenic stroke, it allows for the definition of the stroke etiology in a significant percentage of them, often revealing findings compatible with vasculitis or complicated atherosclerotic disease [9].

Therefore, VW-MRI has become an essential tool for clinicians in the field of cerebrovascular diseases, and continuous technological advances have made it more and more available and reliable for clinical applications.

In this paper we will review the technical basis and the main clinical application of VW-MRI in cerebrovascular disease.

## 2. Acquisition

VW-MRI requires sequences with multiple signal weightings, multiplanar 2D acquisitions or 3D acquisitions, high spatial resolution (HR) and suppression of luminal blood signal [10]. Additionally, sequences for intracranial VW-MRI need the suppression of signal of cerebrospinal fluid (CSF) and extended brain coverage because the site of cerebral vascular pathology is less predictable and defined than the extracranial compartment. The 3D acquisition with isotropic voxels allowing multiplanar reconstruction (MPR) is of utmost importance in the intracranial compartment, where vessels have higher tortuosity, to avoid partial volume artifacts [11].

Multi-contrast VW-MRI is needed for the differential diagnosis of vasculopathies and in the evaluation of plaque composition [12]. Therefore, the MR protocol should include HR 2D or 3D T1 weighted sequence, acquired pre- and post- gadolinium injection, and T2 weighted sequences. Proton density (PD) weighted imaging provides a higher signal-to-noise ratio (SNR) and can be a valid alternative to T1 weighted images. On the other hand, in PD sequences the CSF signal is similar to vessel wall signal, and the contrast enhancement is less evident [4]. The T2 weighted sequence is additional, and it is usually acquired in cases of suspected atherosclerosis [13]. Furthermore, a time-of-flight (TOF) magnetic resonance angiography (MRA) allows for the evaluation of luminal vessel contour and caliber in addition to the vessel wall. The TOF MRA helps the localization of the site of vessels affected and drives the positioning of the VW-MRI sequences. In patients with severe luminal narrowing, contrast enhanced MRA can help to carefully define the actual lumen as low flow velocity may cause flow artifacts within the lumen of the vessel [14]. The MR protocol for VW-MRI is summarized in Table 1.

The use of 3D sequences with MPR reduces the overall MR examination time, avoiding multiple 2D acquisitions in different planes [4]. On the other hand, 2D sequences may provide higher in-plane resolution (up to 0.4 mm of voxel size) than 3D sequences and are of interest when targeting the VW-MRI study to a specific vessel. Therefore, the use of both 3D and 2D images should be considered in VW-MRI protocols. Furthermore, the advent of higher magnetic fields and the use of multichannel coils (32–64 channels) enabled an increased spatial resolution in reasonable acquisition time, even for peripheral vessels, due to the higher SNR [15,16].

In VW-MRI blood suppression techniques are needed to obtain black blood (BB) MR sequences with null signal within the vessel lumen and to avoid flow artifacts that may mimic vessel wall abnormalities. There are several methods to suppress the blood signal: spin echo sequences with a spatial pre-saturation band exploit the movement of blood spin [17]; double inversion recovery (DIR) sequences use both the T1 properties of blood and the blood flow. Disadvantages of DIR sequences are that they are prone to flow artifacts and have long acquisition time. With 3D sequences the main mechanism used to saturate the signal of blood is intravoxel dephasing: the most common 3D BB sequences used in VW-MRI are turbo spin echo (TSE) sequences with variable flip angle refocusing pulse [18]. The names of these sequences vary according to the vendor: SPACE (sampling perfection with application-optimized contrasts using different flip angle evolution, Siemens), CUBE (General Electrics), VISTA (volume isotropic turbo spin-echo acquisition, Philips Healthcare).

In VW-MRI the CSF suppression techniques are useful for a proper outer wall boundary evaluation and are particularly important when studying the peripheral branches of intracranial arteries [19]. Delayed alternating with nutation for tailored excitation (DANTE) preparatory pulse and anti-driven-equilibrium (ADE; Philips Health) or restore (Siemens) sequences suppress CSF signals [20,21].

## 3. Clinical Applications

### 3.1. Atherosclerosis

Atherosclerosis of cervical and intracranial arteries is a common cause of ischemic stroke [22,23]. The atheromatic plaque progression and its surface fissuring leads to cerebral tissue infarct through artery-to-artery embolization, in situ thrombosis, hemodynamic impairment or branches occlusion [3,24]. Current guidelines rely on plaque stenosis severity and symptoms for stratifying the stroke risk and for selecting patients for the best medical therapy or interventional treatments [25,26]. However, the stroke risk is only partially explained by stenosis degree, and some patients experience ischemic stroke despite subcritical stenosis [27]. These evidences and histopathological studies support the concept that other markers of plaque vulnerability, such as intraplaque hemorrhages (IPH), surface irregularity and ulceration, dimension and structure of fibrous cap and lipid core [28], may help in the stroke risk stratification. VW-MRI could overcome the limitations of lumen-based imaging techniques [28,29], and improve the etiological workup of ischemic stroke, through the characterization of atheromatic plaque and the differential diagnosis of vessel wall abnormalities and vasculopathies [27,30].

This section summarizes the role of VW-MRI for intra and extracranial vessels study.

The use of conventional imaging for the recognition and phenotyping of the atheromatic plaque and stenosis quantification in intracranial arteries is hampered by the small vessel size [23].

The VW-MRI features of the atheromatic plaque are the presence of focal, eccentric wall thickening, with hyperintensity on T2W sequences and wall enhancement on T1W postcontrast sequences [4]. Although this district the spatial resolution often limits the identification of single plaque layers, the external fibrous cap (FC) may be distinguished from the underlying lipid core. The former appears hyperintense on T2W sequences and iso-hyperintense on (PD) sequences, the latter hypointense on T2W and PD sequences and isointense on T1W sequences. Dot-like hypointensities on T1W and T2W sequences are typically suggestive of calcifications [31]. These features on VW-MRI differentiate the atheromatic plaque from other intracranial non-stenotic vasculopathies whit higher accuracy than other luminal modalities [32,33].

Moreover, VW-MRI improves the quantification of stenosis severity, with a similar performance when compared to digital subtraction angiography (DSA), which remains the gold standard technique [31,33], and it provides important information about the activity of the atheromatic plaque.

Plaque enhancement on T1W postcontrast sequences is a hallmark of symptomatic plaque and reflects intralesional inflammation and neovascularization [34]. The enhancement pattern is often diffuse or, rarely, layered shape, with distinct enhancement of the fibrous cup and the adventitia [4]. The more intense is the plaque enhancement, the greater is the probability of a causal relationship between the plaque and the ischemic event [35]. In a prospective cohort study employing 7T VW-MRI, Fakih et al. demonstrated that a plaque-to-pituitary stalk contrast ratio (CR) greater than 0.53 is an independent predictor of culprit plaque, with a sensitivity of 78% and a specificity of 62% [35]. Furthermore, plaque enhancement represents an independent predictor of stroke recurrence [34,36]. To avoid misdiagnosis, it is worth noting that T1W concentric enhancement of vessel wall may be detected after endovascular treatment [37,38].

Symptomatic plaques seem thicker than asymptomatic ones and have a higher degree of surface irregularity [4,36]. Another evidence of plaque activity is vessel remodeling. The positive remodeling is a compensatory external bulging of the vessel wall, likely arising from intraplaque hemorrhage and inflammation; conversely, the “negative remodeling”, is the restriction of the vessel size, which reflects fibrosis. The former, as a marker of plaque vulnerability, is associated to symptomatic plaques and to an increased risk of ischemic events [31,36,38].

A further marker of high-risk plaque is the IPH. Its VW-MRI features are mostly derived from extracranial studies and reflect the oxidative state of hemoglobin. Acute IPH is hyperintense on T1W sequences and it is defined as an intraplaque area of high signal, with intensity greater than 150% of the signal of adjacent muscles [39] or adjacent grey matter [40]. The association between intracranial IPH plaque and an increased risk of stroke may be inferred from carotid artery studies [28], but this relation is not well established for intracranial vessels so far [38].

Although the greater dimension of arteries in the extracranial district makes easier the recognition of atheromatic plaque and its features with traditional techniques, the role of VW-MRI is relevant likewise to explore plaque vulnerability [29].

VW-MRI displays plaques’ surface and distinguishes three types of plaque: regular, irregular, and ulcerated (with cavities depth > 1 mm), which is strongly linked with risk of thromboembolism. Its sensitivity is similar to CTA [29], and it provides thickness quantification alike to ultrasound (US) and computed tomography angiography (CTA) [28].

The main strength of VW-MRI in this context is the proper detection of different plaque components (Table 2). It is the best technique to detect IPH and its evolution status, with greater accuracy than US and CTA [28]. In addition, VW-MRI performs better than CTA in the detection of the lipid-rich necrotic core (LRNC) and the FC. A thin, irregular FC and its ulceration are markers of stroke risk, and its rupture leads to exposure of LRNC to blood flow. The dimension of LRNC, in turn, correlates with the disruption of FC and cerebrovascular events [41].

Neovascularization and inflammation are markers of plaque activity that correlate with the degree of enhancement [28].

Most of the available evidence derives from anterior circulation imaging, whereas the role of VW-MRI in posterior circulation atherosclerosis remains poorly defined.

To summarize, VW-MRI may provide additional diagnostic value in atheromatic plaque characterization and stratification of stroke risk in clinical practice, and it is a valuable diagnostic aid in patients with cryptogenic stroke and suspected intracranial atherosclerotic disease (ICAD). In Fakih et al. contribution, 7T VW-MRI allowed the etiology of stroke in 28/34 (82.4%) patients with cryptogenic stroke and clinically suspected intracranial vasculopathy to be defined, 89.3% of whom were diagnosed with ICAD [35].

### 3.2. Vasculitis

Central nervous system (CNS) vasculitis is a rare cause of stroke, affecting younger patients and also children [42]. Vasculitis-involving CNS can be divided into primary (primary angiitis of central nervous system, PACNS), and secondary vasculitis associated with systemic infectious or autoimmune diseases [42,43]. Focal neurologic signs (63%), headache (51%) and cognitive impairment (41%) are the most frequent symptoms at onset; less frequently seizures, fever or elevated inflammatory markers are present [44].

Diagnosis of CNS vasculitis is challenging, particularly in PACNS, and DSA and brain biopsy have a very low concordance, as DSA is positive in 33% of biopsy confirmed cases, and biopsy was positive in 8% of angiogram confirmed cases [44]. MRI shows high sensitivity (97–98%), but specificity remains low [44]. Common findings on MRI are ischemic lesions, frequently bilateral and multiple, leptomeningeal or parenchymal contrast enhancement and less frequently intraparenchymal hemorrhage [45]. DSA is the preferred diagnostic modality for luminal imaging in PACNS, revealing smooth-wall segmental narrowing or enlargement and multifocal stenosis of cerebral arteries, but these findings are present in only 61–64% of patients, and even less frequently on MRA [46,47]. Moreover, the luminal vessel abnormalities, like MRI findings, are often nonspecific, and in many cases can mimic either atherosclerotic disease or noninflammatory intracranial vasculopathies like reversible cerebral vasoconstriction syndrome (RCVS). VW-MRI can detect in vivo the inflammatory alterations of intracranial arteries vessel walls linked to vasculitic processes, independently from their stenosing effect, helping in the differential diagnosis of CNS vasculitis [4,5,48,49].

The hallmark of CNS vasculitis on VW-MRI is concentric vivid vessel wall enhancement (VWE) of intracranial arteries, often multifocal, with variable associated luminal stenosis and wall thickening [7,12]. In a recent systematic review, imaging features of intracranial vasculitis affecting the intracranial and extracranial arteries were VWE (89%), vessel wall thickening (72%), vessel wall edema (10%) and perivascular enhancement (16%) [50]. The pattern of enhancement of vasculitis lesions were concentric in the majority of cases and less frequently combined concentric and eccentric; only the eccentric patter was present in 7% of patients [50]. Perivascular enhancement has been reported in infectious vasculitis such as Varicella–Zoster virus (VZV) and tuberculosis associated cases [50].

Beside PACNS, VW-MRI can depict inflammatory changes in a wide range of secondary vasculitis, including radiation-induced and those associated with infectious disease as the human immunodeficiency virus (HIV), syphilis and VZV [51,52,53]. Preliminary findings obtained with VW-MRI also suggested a possible inflammatory mechanism underlying a percentage of cryptogenic stroke in Coronavirus disease 2019 (COVID-19) patients [54,55]. So, including VW-MRI in a diagnostic workup of patients with cryptogenic stroke, particularly if an inflammatory etiology is suspected, may provide additional diagnostic yield [7].

Another application of VW-MRI is to target brain biopsy when considered essential for diagnosis. In this scenario it can increase sensitivity to 89% from a baseline performance as low as 36% detected in previous studies [56]. Some authors even suggested that in selected patients, particularly in those with suspected secondary CNS vasculitis, if clinical data are consistent, it may allow brain biopsy to confirm the disease to be avoided [57].

**Figure 1:**
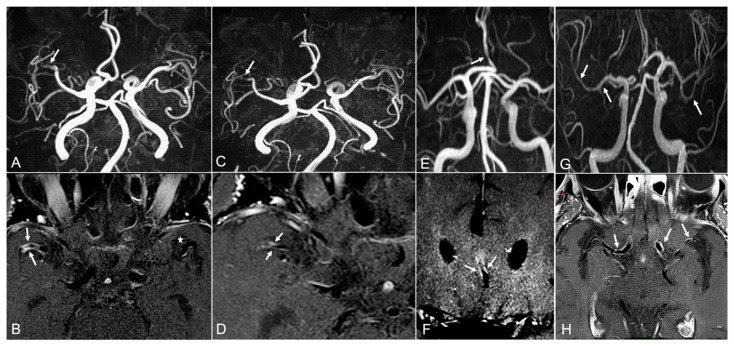
VWMRI findings in CNS vasculopathies. TOF-MRA of a patient with PACNS and recurrent ischemic strokes shows stenosis of M1 segment of MCA (panel (**A**), white arrow) with corresponding vivid concentric enhancement of arterial walls in post contrast in T1W black blood sequence (panel (**B**), white arrows) (* contralateral normal M1 segment). After treatment with intravenous high dose methylprednisolone and cyclophosphamide bolus, contrast enhancement was markedly reduced (panel (**D**), white arrows) with stable MRA findings (panel (**C**), white arrow). The patients had no recurrence of stroke during follow-up. MRA and VW-MRI findings of a patient with COVID19-associated cryptogenic ischemic stroke demonstrated stenosis of ACA (panel (**E**), white arrow) with corresponding contrast enhancement on VW-MRI (panel (**F**), white arrow). MRA of a patient with syphilitic CNS vasculitis revealed multifocal arterial lumen irregularities (panel (**G**), white arrows) and VW-MRI demonstrated multifocal enhancement of correspondent MCA segments (panel (**H**), white arrows). CNS: central nervous system; TOF: time of flight; MRA: magnetic resonance angiography; PACNS: primary angiitis of the CNS; MCA: middle cerebral artery; T1W: T1 weighted; VWMRI: vessel wall MRI; COVID19: Coronavirus disease 2019.

Main VW-MRI findings in CNS vasculitis compared and other intracranial vasculopathies are summarized in Table 3, and examples of VW-MRI images are depicted in Figure 1.

In giant cells arteritis (GCA), VW-MRI allows direct identification of the inflammatory changes in frontal and parietal branches of the superficial temporal artery and of the occipital artery. Presence of mural thickening (>0.6 mm) and significant mural enhancement confirm the diagnosis [58]. Using 3D T1W contrast-enhanced sequences, VW-MRI demonstrated 80% sensitivity and 100% specificity for detecting GCA, performing better than temporal artery biopsy [59]. Moreover, in patients presenting with anterior ischemic optic neuropathy (AION), VW-MRI can distinguish between arteritic and non-arteritic etiology. In a prospective study, VW-MRI identified inflammatory changes of the ophthalmic artery in 100% of patient with arteritic AION; other associated findings were enhancement of the optic nerve sheath (82%) and inflammatory changes of the posterior ciliary arteries (82%) or extracranial arteries (77%) [60].

In CNS vasculitis VW-MRI can also be used to follow-up patient during immunosuppressive therapy. In a prospective study, VWE regressed in seven of nine PACNS patients after treatment, at a median follow-up period of 8 months, with three of these who also showed regression of vessel stenosis [61]. Persistent or progressive VWE is associated with a higher risk of relapses in patients with PANCS and secondary CNS vasculitis [62]. Nevertheless, persistent VWE must be interpreted cautiously because it can be of uncertain clinical significance and can be seen in patients with clinical remission even at long term follow-up. In these cases, it probably represents post-inflammatory arterial wall changes, like mural fibrosis with or without neovascularization [62].

### 3.3. Intracranial Dissections

Intracranial artery dissections (IAD) are an uncommon cause of ischemic stroke and subarachnoid hemorrhage (SAH). They frequently affect young patients without traditional vascular risk factors and in some patients can be the manifestation of connective tissue disease [63]. IAD involve predominantly the anterior circulation in western countries and posterior circulation in eastern countries [64]. Supraclinoid internal carotid artery is the most frequently affected vessel of anterior circulation and vertebral artery the most affected segment in posterior circulation. Less frequently, other intracranial arteries can be involved. IAD may be subsequent to an intimal tear or caused by an intramural hemorrhage without communication to the vessel’s lumen [65,66].

The main diagnostic radiological findings of IAD on luminal imaging include fusiform or irregular aneurysmal dilation at a non-branching site, long irregular stenosis associated with double lumen, rapid morphologic change on repeat imaging, focal stenosis and dilation (“string and pearl sign”) or arterial occlusion with recanalization at a non-branching site with fusiform or irregular aneurysmal dilation [67]. DSA has been the standard diagnostic procedure for suspected dissection, but because it can’t directly visualize the vessel wall, it does not always ensure definitive diagnosis of dissection.

Pathognomonic features of IAD are intramural hematoma, intimal flap and double lumen, and all can be identified using VW-MRI [3]. In a recent meta-analysis, the reported frequency of intramural hematoma, vessel wall enhancement, aneurysmal dilatation, and intimal flap/double lumen sign differs, being, respectively, 86%, 75%, 71% and 47% [68].

Intramural hematoma appears as an eccentric focal thickening of the arterial wall, with a crescent shape. The signal varies depending on the temporal stage. It becomes spontaneously hyperintense on T1-weighted sequences 48–72 h after onset for the presence of methemoglobin. And its recognition is higher in the late subacute stage of dissection than in acute and early subacute stages. [69] T2-weighted sequences may contribute to define the temporal stage of hematoma. The acquisition of T2*-weighted or susceptibility weighted MR imaging may allow for an earlier detection of intramural hematoma, due to blood deoxygenation, even before it is hyperintense in T1- weighted sequences [4].

Aneurysmal dilatation of the outer wall can occur and should be evaluated measuring the diameter of the vessel at the perpendicular plane, at the level of the lesion. [69] Its evaluation is important, because intramural hematoma can be difficult to visualize in the acute and early subacute stages, particularly in smaller vessels. In one study, aneurysmal dilation was identified in 80% of anterior cerebral artery (ACA) dissection in the acute stage (0–3 days), and in 100% of the cases in the early subacute stage (3–10 days) using BB T2 weighted sequences, even in cases in which intramural hematoma was not detectable on T1 weighted images in that phases of the disease [69]. Similar results have been obtained with PICA dissection in acute and early subacute phases [70].

Direct visualization of the intimal flap and/or double lumen is the most significant finding but the least frequently detectable. The intimal flap is visualized as a layer crossing the arterial lumen; it can be seen as a linear layer of low signal intensity in the context of a high signal hematoma in T1W pre-contrast sequences, as a linear enhancing layer on post-contrast T1 images or as a curvilinear hyperintensity in T2W sequences [63,71].

In post contrast T1 weighted sequences, focal enhancement of the arterial wall can be present in IAD, probably secondary to slow blood flow in the false lumen, inflammation, and enhancement of the vasa vasorum [4].

The differential diagnosis of IAD presenting with focal stenosis or complete occlusion is sometimes difficult, particularly in middle cerebral artery (MCA) dissection, as intramural hematoma and intraplaque hemorrhage (IPH) may look similar. Therefore, integration with clinical information, particularly focusing on vascular risk factors and follow-up imagining are mandatory [72]. In vertebral artery dissection, T2 weighted BB sequences helped in differential diagnosis. Dissection of false lumen shows a lower signal intensity than IPH on BB T2-weighted imaging and false lumen has n higher signal intensity than IPH on TOF images [73].

IAD are dynamic lesions and changes in appearance are present in more than 80% of cases in the first two weeks after dissection and can continue for months. Complete recanalization is seen in 20% and, overall, at 6-month recanalization is seen in 20–58% of patients, while 30–77% have no change in vessel lumen [65].

### 3.4. Reversible Cerebral Vasoconstriction Syndrome

RCVS is characterized by the multifocal vasoconstriction of intracranial arteries with consequent stenosis. The pathogenesis is thought to be a transient alteration of cerebral vasculature tone regulation. Clinically, thunderclap headache, often recurrent over one or two weeks, is the most common symptom; ischemic and hemorrhagic stroke are the main complications [74,75]. RCVS is a monophasic disease with a low recurrence rate and relapses are mostly benign [76]. Triggers as RCVS postpartum state, pre-eclampsia, recreational drugs, vasoactive substances, antidepressants, blood transfusions can be identified in some patients [77].

DSA is considered the gold standard to differentiate RCVS from CNS vasculitis and intracranial atherosclerosis and shows multifocal narrowing of intracranial arteries, that usually reverse in less than three months from onset. The dynamic nature of the stenosis is a cardinal feature for the diagnosis of the syndrome and can be appreciated even at close follow-up during the acute phase. Stenosis involves more frequently MCA, ACA and posterior cerebral artery (PCA); conversely, involvement of intracranial segment of the internal carotid artery is less frequent and usually point to another diagnosis as Moyamoya and intracranial atherosclerosis. The posterior circulation arteries are often involved and the abnormalities are more diffuse in RCVS then in other intracerebral arteriopathies [78]. However, DSA in an invasive procedure, which can carry the risk of serious complications, and so it is not ideal for serial imaging required in highly dynamic disease as RCVS.

MRI can detect luminal stenosis with an 80% sensitivity compared to DSA and can contextually evaluate brain parenchyma for the main RCVS-associated complications that are important for therapeutic purposes. Associated imaging findings on MRI in RCVS patients are convexity SAH, posterior reversible encephalopathy syndrome (PRES)-like lesions, ischemic stroke, intraparenchymal hemorrhage and hyperintense cortical vessel on FLAIR images [78,79].

Even if DSA has a high sensitivity in detecting luminal stenosis in RCVS, the pattern and distribution of these lesions significantly overlap with vasculitis lesions (mainly PACNS), and differential diagnosis can be challenging, especially at presentation, when longitudinal follow-up imagining is not available. Moreover, misdiagnosis of RCVS with a vasculitic process can expose the patient to unnecessary and harmful immunosuppressive treatment that can precipitate the underlying disease [80,81].

In this context, VW-MRI can have an important role in discriminating between RCVS and its mimics [82].

Intracranial VW-MRI findings in RCVS are multifocal stenosis with concentric iso- or hypo-intense wall thickening, often non-enhancing (≅55%), or less frequently with mild concentric enhancement (≅35%), that can rarely be more marked (≅10%) [83,84]. Enhancement, if present, is clearly less intense that in vasculitis and the concentric thickening whit no signal heterogeneity, the absence of positive remodeling and eccentric enhancement distinguish RCVS from atherosclerotic lesions [32,83,84].

At follow up, vessel wall enhancement and arterial wall thickening are expected to completely disappear in the majority of patients, but 10% of them can show persistent mild enhancement [82]. o the absence of complete resolution of wall enhancement does not rule out the diagnosis of RCVS, and, at least in some cases, can be secondary to underlying intracranial atherosclerosis, particularly in patients with known vascular risk factors [85].

### 3.5. Moyamoya Disease and Moyamoya Syndrome

Moyamoya disease (MMD) is a progressive non-atherosclerotic, non-inflammatory occlusive disease, involving distal internal carotid (ICA) arteries and large intracranial arteries of anterior circulation. The consequent development of collaterals at the skull base is responsible for the “puff of smoke” angiographic appearance, that gives the name to the disease. Etiology is unknown and histological examination shows hyperplasia proliferation of smooth muscle cells, particularly involving the tunica media associated with an irregular elastic lamina [86,87].

Moyamoya syndrome (MMS) is characterized by similar progressive stenosis involving the same vascular territory, but secondary to an underlying disease; the differential diagnosis of the underlying cause includes sickle cell anemia, neurofibromatosis 1, radiation therapy, congenital syndromes, intracranial atherosclerotic disease and intracranial vasculitis. The differentiation between MMD and MMS is critical, because the treatment is different and some of the underlying disease have a specific and effective therapy [88].

The diagnosis relies usually on luminal imaging, CTA or DSA. However, the pattern of luminal stenosis is very similar between MMD and MMS. VW-MRI can discriminate between different etiologies, directly depicting the pathologic process involving arterial walls [89,90,91].

In MMD, VW-MRI reveals ICA and middle cerebral arteries diameter shrinkage (ICA/basilar artery ratios < 1) with associated luminal stenosis. Vessel walls thickness can vary during the disease and can be normal or increased, with homogeneous T2 signal. Contrast enhancement can be either absent or mild, concentric, and homogeneous, involving distal internal carotid arteries and middle cerebral arteries; it can be bilateral. MMS related to vasculitic processes (V-MMS) presents with radiological findings of vasculitis, demonstrating vivid concentric enhancement of arterial wall, without positive remodeling or T2 signal inhomogeneity. Atherosclerotic MMS (A-MMS) can be diagnosed identifying the radiological characteristics of intracranial plaques as eccentric lesions with T2 heterogeneous signal and possible focal nonconcentric contrast enhancement, particularly in active symptomatic plaques [88,92,93].

In a retrospective study, there was a statistically significant improvement in overall diagnostic accuracy from 32% to 87%, respectively, from 37% to 90% in MMD, from 13% to 100% in vasculitic MMS and from 32% to 82% in atherosclerotic MMS [88].

Aside differential diagnosis, vessel wall enhancement of steno-occlusive lesions in patient with MMD has been associated with intracranial hemorrhagic episodes [94] and increased risk of new ischemic lesions at 4 week follow-up [9].

### 3.6. Intracranial Aneurysms

Intracranial aneurysms are the leading cause of non-traumatic (SAH) [95]. Unruptured intracranial aneurysms (UIA) are common lesions, affecting 3–5% of adults and they have a low baseline yearly risk of rupture. The decision to treat asymptomatic intracranial arteries aneurysm is often challenging, and depends on both patient’s and lesions characteristics [96]. The most important aneurysmal determinants of annual rupture risk are size, shape, location and evidence of growth over time [97,98].

Inflammatory activity in the aneurysmal wall, neovascularization and atheromatic lesions inside the aneurysmal sac are key processes that leads to aneurysm wall fragility, and eventually to enlargement and rupture [99].

VW-MRI may depict these processes and aneurysm wall enhancement (AWE) extent has been associated with the degree of histological inflammatory markers, as well as atherosclerosis, neovascularization, and macrophage infiltration [8,99,100].

VW-MRI may provide an added value in estimating the rupture risk and AWE is the most important VW-MRI finding associated subsequent SAH [4,101]. AWE can be assessed qualitatively or quantitatively. Despite different methods of quantitative assessment exist, in recent study aneurysm-to–pituitary stalk contrast ratio using maximal signal intensity was the most reliable one [102].

In several studies AWE was associated with UIA instability [103] and an increased risk of enlargement, particularly with daughter sac formation, and rupture [104,105]. The absence of AWE showed a strong negative predictive value and sensitivity for aneurysmal rupture and no SAH during follow-up, that reached, respectively, 96.2% and 95% [105].

The absence of AWE, integrated with known risk factors, may be useful to select patients with unruptured aneurysm who are suitable for a conservative approach. Specificity and positive predictive value were much lower, respectively 62.7% and 55.8%, and AWE should be interpreted only along with other known independent risk factors and patients’ characteristics to define an UIA at high risk of rupture before deciding for treatment [105].

Another application of VW-MRI is the differentiation between intradural and extradural of unruptured paraclinoid aneurysms of ICA. This categorization is important for both prognostic information and therapeutic decisions, as intradural paraclinoid aneurysm have a significant risk of rupture with consequent SAH. In contrast, extradural paraclinoid UIA usually have a benign course and become symptomatic of mass effect on adjacent structures, but in case of rupture in the cavernous sinus, a carotid cavernous fistula may develop. In a retrospective study, VW-MRI was superior to DSA/MRA in the definition of the intra- or extradural location of paraclinoid ICA aneurysms, with a classification rate of 80% and 47%, respectively [106].

In the context of a recent aneurysmal SAH, VW-MRI may provide additional information for the acute management. First it can help to identify the culprit aneurysm in presence of multiple lesions—in fact AWE is more common in recently ruptured and symptomatic aneurysm than in asymptomatic ones [107]. Second, it may be able to predict the risk of occurrence of intracranial artery vasospasm. In fact, concentric arterial wall enhancement in patients with recent SAH had been related to the subsequent development of vasospasm in the involved arterial segments [108].

Lastly, in patients with nontraumatic SAH and normal DSA, which account for 15–20% of cases, VW-MRI revealed abnormal findings in 82.4% of cases, suggesting the utility of this technique in this setting. Abnormalities included intracranial artery dissections, blood blister-like aneurysms, ruptured fusiform aneurysms and focal nodular wall enhancement [109].

However, despite the promising data, larger longitudinal studies must confirm these preliminary findings before they can routinely be used in clinical practice.

### 3.7. Brain Arteriovenous Malformations

Brain arteriovenous malformations (AVMs) consist of a network of abnormal blood vessels with a direct connection between arterial feeders and veins, without the interposition of capillaries. The main complication of AVMs is rupture, with consequent intracranial hemorrhage, and often seizures [110]. Clinical management of patients with unruptured AVMs is guided by the balance between rupture risk and treatment-related risks. The main treatment options are embolization, surgical resection, and stereotactic radiosurgery. The complete obliteration of the AVM nidus is the optimal goal, but not always feasible and carries significant risks [110,111]. The annual risk of hemorrhage of unruptured AVMs is around 1% year, but is influenced by many factors as associated aneurysms, deep location, deep drainage and increasing age [111]. The risks associated with treatment and the uncertain benefit in the short term in unselected patients, especially after results of ARUBA trial [112], make patients’ management challenging. A better stratification of patients is needed to identify those with high AVM rupture risk, who would benefit from interventional treatment [113].

VW-MRI has been used to assess the rupture risk in UIA and to identify the culprit lesion in the presence of multiple IA in patients with SAH. The same approach has been proposed for AVMs. Particularly, VW-MRI can identify the site of the bleeding inside the AVM nidus showing VWE of the involved vascular structure and adjacent blood products; moreover, it can help to exclude a high-risk vascular structure as the source of bleeding. These data can be crucial if a targeted partial embolization of the AVM is planned to prevent rebleeding [114].

However, this approach is based on a few case series [114,115] and case reports [116,117,118] and in unruptured AVM, a retrospective study of nine patients revealed a very high prevalence of VWE (8/9), questioning its utility in identifying high rupture risk malformations. Authors also suggested that VWE may be in some case related to incomplete blood suppression due to the complex hemodynamic of AVM [119].

Overall, there is a scarcity of available data, and previous studies on the use of VW-MRI in AVMs have important limitations, and no strong conclusion can be drawn [119]. More research is needed before this technique can be confidently used to assess patients with AVMs in clinical practice.

## 4. Pitfalls

In VW-MRI misinterpretations of findings derive mainly from technically incomplete blood signal suppression and from conditions that may mask or mimic vessel wall enhancement.

Incomplete blood signal suppression in VW-MRI sequences may mimic an eccentric or concentric vessel wall thickening. Artifacts due to incomplete blood signal suppression are more frequent where blood flow is reduced, recirculating or disturbed; in vessels that are curved and of large diameter (e.g., the genu of petrous ICA or the cavernous segments of ICA), proximal or distal to a stenosis or in aneurysms [14,120]. Additionally, the cerebral blood flow is reduced in the periphery of the vessel lumen due to the laminar distribution of the blood velocity, which is lower nearby the vessel wall, where, as a consequence, flow artifacts are more frequent [121]. Also, MRA without gadolinium can be sensitive to the same mechanism that causes flow artifacts in VW-MRI sequences, thus MRA with the injection of gadolinium may help in avoiding misinterpretation of flow artifacts demonstrating the lumen patency [14]. Attention should also be paid to the orientation of slices as the flow suppression is most effective when the acquisition plane and the read-out encoding frequency are parallel to the vessel orientation [120]. Therefore, the acquisition of VW-MRI sequences in multiple planes may avoid artifacts as multiple vessels will be imaged in the proper plane.

Several factors may mimic a VW enhancement. The parenchymal enhancement of subacute ischemic stroke, due to the disruption of blood brain barrier, may appear as an inflammatory enhancement of a small parenchymal vessel. The use of diffusion weighted imaging (DWI) helps in depicting the area of ischemia [14]. Moreover, in acute stroke patients treated with mechanical thrombectomy, VWE often occurs, representing endothelial damage, inflammation and greater disruption of the blood–brain barrier caused by devices, and it is more pronounced with the use of stent retrievers, than aspiration catheters [37].

The central hypointensity of microhemorrhages with the surrounding enhancement due to the inflammatory response of the adjacent tissue [122,123] may mimic the flow-suppressed vessel lumen with VW-MRI enhancement. The use of susceptibility weighted imaging (SWI) and 3D imaging help to distinguish the single dot of hypointensity of the microbleed from the linear hypointensity of a vessel lumen [14].

The enhancement of the vascular venous plexus surrounding the arteries at the skull base can be misinterpreted as vasculitis or atherosclerotic changes. A common site of this misinterpretation is the petrous segment of the internal carotid artery [124]. Similarly, the vasa vasorum present in VW of arteries when they enter in the intracranial compartment may mimic VW enhancement, as commonly seen in the V4 segment of the vertebral artery [125]. Additionally, veins have a high density of vasa vasorum responsible for the vessel wall enhancement even in absence of a pathological process [126].

Leptomeningeal enhancement can be misinterpreted as a perivascular inflammation. Multiple oblique planes of reconstruction of 3D VW-MRI may clarify the distribution of the enhancement and help to distinguish leptomeningeal from perivascular enhancement [56].

Finally, false negatives may arise from the use of steroid therapy that can attenuate the degree of enhancement reducing the inflammatory activity [127]. Additionally, an inadequate delay after gadolinium injection, that should be at least of five minutes, may results in a lack of visualization of contrast enhancement [128].

Major pitfalls for the interpretation of VW-MRI are summarized in Table 4.

## 5. Conclusions

VW-MRI has become a valuable aid in investigating patients with acute cerebrovascular disease and shows a wide range of clinical applications. Recent advances in MRI technology and the diffusion of high magnetic field devices made VW-MRI widely available. Its value in the differential diagnosis of intracranial vasculopathy is already recognized, and VW-MRI may be included in the diagnostic workup of several patients, as it provides adjunctive information not obtainable with traditional luminal-based imaging techniques [3,125]. Further applications, like risk stratification in patients with intracranial atherosclerotic disease and intracranial aneurysms, offering the potential to tailor the best therapeutic approach in fields is of great interest, but it requires larger prospective studies [129]. Furthermore, the introduction of 7T MRI devices in clinical practice may further increase the possibility of VW-MRI, allowing for better signal-to-noise ratios and a better definition of intracranial lesions [130,131,132].

## Figures and Tables

**Table 1:** Principal magnetic resonance sequences used for vessel wall magnetic resonance imaging (VW-MRI).

MR Sequences	Technical Requirements	Contrast Medium	Findings
T1-weighted (or PD) sequence	High spatial resolution; multiplanar 2D or 3D acquisition; blood and CSF signal suppression	Before and after Gd iv administration	Depiction of VW enhancement
T2-weighted sequence	High spatial resolution; multiplanar 2D acquisition	No need of Gd iv administration	Additional; usually acquired in cases of suspected atherosclerosis
MRA	Extended brain coverage; MIP reconstructions.	With or without Gd iv administration	Depiction of the site of vascular pathology; consider CEMRA in case of severe arterial narrowing or dilation

PD: proton density; CSF: cerebrospinal fluid; Gd: gadolinium; i.v.: intravenous; VW: vessel wall; MRA: magnetic resonance angiography; MIP: maximum intensity projection; CEMRA: contrast enhanced MRA; T1-w: T1-weighted.

**Table 2:** Characteristics of atheromatic plaque components on different VW-MRI sequences [3,31].

Plaque Component	3D TOF	T1W	T2W	PD	GdT1W	Clinical Significance
Fibrotic tissue (1)	Iso	Iso/Hyper	Iso/Hyper	Iso/Hyper	Yes	Thin/ruptured FC is associated with higher risk of stroke
Lipid core (2)	Iso	Iso/Hyper	Hypo	Iso/Hyper	No	Increasing LRNC is associated with FC rupture, and plaque vulnerability
Calcifications (3)	Hypo	Hypo	Hypo	Hypo	No	
Hemorrhage (4)						
Acute (<1 week)	Hyper	Hyper	Iso/Hypo	Iso/Hypo	No	IPH is associated
Subacute (1–6 weeks)	Hyper	Hyper	Hyper	Hyper	No	to plaque progression
Chronic (>6 weeks)	Hypo	Hypo	Hypo	Hypo	No	
	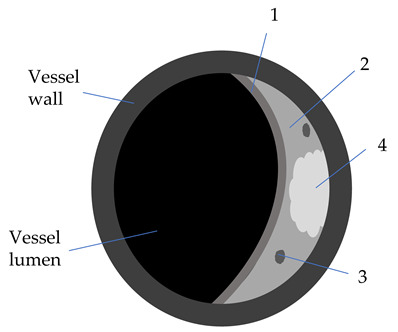	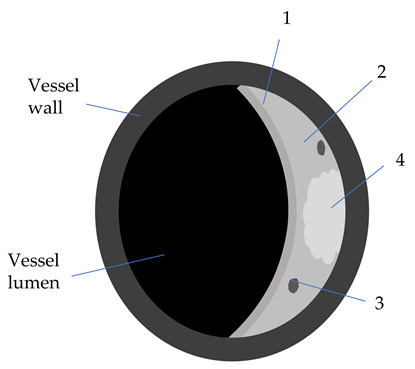	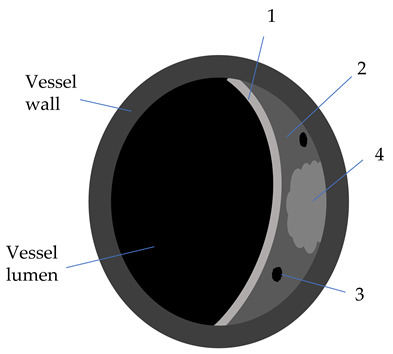	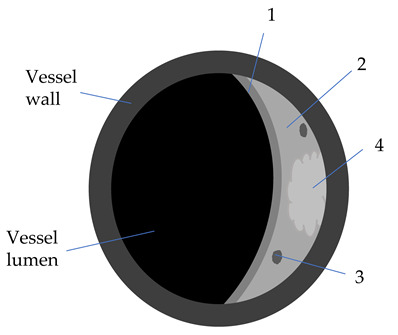	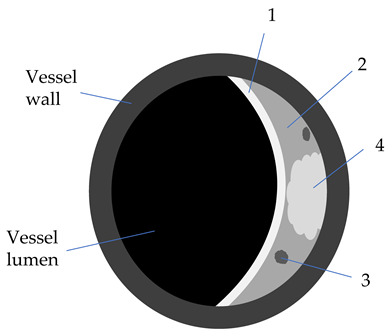	

3D-TOF: 3D-time of flight; T1W: T1 weighted; T2W: T2 weighted; Gd: gadolinium; Hyper: hyperintense; Hypo: hypointense; FC: fibrous cap (1); w: weeks; LRNC: lipid-rich necrotic core (2); IPH: intraplaque hemorrhage (4); calcifications (3).

**Table 3:** Main VWMRI findings in intracranial vasculopathies.

	Vasculitis	Atherosclerosis	RCVS	IAD	MMD	A-MMS	V-MMS
**Vessel wall**	Thickened, concentric.	Thickened eccentric.	Thickened concentric.	Thickened eccentric with signal characteristic of blood products (intramural hematoma).	Thickened or normal.	Thickened, concentric.	Thickened, eccentric.
**Contrast enhancement**	Vivid, concentric, homogeneous.	Variable, present in active plaques; eccentric.	Absent or mild concentric.	Focal enhancement +/−	Absent or mild concentric (the latter associated with subsequent ischemic or hemorrhagic events).	Variable, present in active plaques; eccentric.	Vivid, concentric, homogeneous.
**Vessel lumen**	Stenosis, often multifocal.	Variable degree of stenosis; may present with positive vessel remodeling without stenosis.	Multifocal stenosis, reversible, posterior circulation often involved.	Luminal stenosis with associated dilatation of outer arterial wall diameter. Presence of intimal flap/double lumen sign.	Progressive stenosis of ICA and proximal MCA. Outer vessel diameter may be reduced.	Like MMD, concomitant atherosclerotic stenosis of other intracranial vessels may be present.	Like MMD but may involve other vessels atypical for MMD.

RCVS: Reversible cerebral vasoconstriction syndrome; IAD: intracranial artery dissection; MMD: Moyamoya disease; V-MMS: vasculitic Moyamoya syndrome; A-MMS: atherosclerotic Moyamoya syndrome; ICA: intracranial carotid artery; MCA: middle cerebral artery.

**Table 4:** Pitfalls for the interpretation of VW-MRI. The causes of pitfalls, the site where pitfalls are more frequently encountered, and the possible solutions are listed.

Pitfalls	Causes	Common Site of Artifact	Improvements
Conditions that may mimic atherosclerotic plaque or VW thickening	Incomplete blood flow suppression at T1w-images	Curved and large diameter vessels (genu of petrous ICA; cavernous segments of ICA); proximal or distal to a stenosis; laminar blood flow close to the VW	Acquisition of VW T1 sequences in multiples planes to increase the number of vessel segments parallel to the frequency-encoding direction to improve blood signal suppression
	Flow artifacts at MRA	Curved and large diameter vessels (genu of petrous ICA; cavernous segments of ICA); proximal or distal to a stenosis	Acquisition of CEMRA to demonstrate proper lumen patency
Conditions that may mimic inflammatory VW enhancement	Parenchymal enhancement of subacute ischemic stroke	Intraparenchymal vessels	DWI helps in depicting the area of ischemia
	Microhemorrhages with surrounding inflammatory response	Intraparenchymal vessels	SWI and 3D imaging help to distinguish the single dot of hypointensity of the microbleed from the linear hypointensity of the vessel lumen.
	Enhancement of the vasa vasorum or of vascular venous plexus	Arteries at their entry in the intracranial compartment, petrous segment of the internal carotid artery and V4 segment of the vertebral artery	3D multiplanar reconstructions parallel to long axis of the vessel or 2D perpendicular to short-axis section of the vessel for higher-spatial resolution to delineate the enhancement from vascular plexus
	Leptomeningeal enhancement	Pial vessels	Multiple oblique planes of reconstruction of 3D VW images may clarify the distribution of the enhancement.
Conditions that may mask inflammatory VW enhancement	Use of steroid	Any vessels	MR scan acquisition before the start of steroid therapy
	Inadequate delay after Gd administration	Any vessels	Acquisition of VW images at least 5 min after Gd administration

PD: proton density; CSF: cerebrospinal fluid; Gd: gadolinium; i.v.: intravenous; VW: vessel wall; MRA: magnetic resonance angiography; MIP: maximum intensity projection; CEMRA: contrast enhanced MRA; T1-w: T1-weighted; ICA: internal carotid artery; DWI: diffusion weighted imaging; SWI: susceptibility weighted imaging.
